# Internalised Stigma, Self-Esteem and Perceived Social Support as Psychosocial Predictors of Quality of Life in Adult Patients with Schizophrenia

**DOI:** 10.3390/jcm13226959

**Published:** 2024-11-19

**Authors:** Corina Gagiu, Vlad Dionisie, Mihnea Costin Manea, Doina Carmen Mazilu, Mirela Manea

**Affiliations:** 1 Department of Psychiatry and Psychology, “Carol Davila” University of Medicine and Pharmacy, 020021 Bucharest, Romania; 2 “Prof. Dr. Alexandru Obregia” Clinical Hospital of Psychiatry, 041914 Bucharest, Romania; 3 Department of Nursing, “Carol Davila” University of Medicine and Pharmacy, 020021 Bucharest, Romania

**Keywords:** internalised stigma, self-esteem, social support, coping, adherence to treatment, age at illness onset, schizophrenia, quality of life, satisfaction with life

## Abstract

**Background:** Schizophrenia is a chronic and severe mental illness that ultimately leads to reduced quality of life (QoL). Over the years, QoL has emerged as an important outcome in the treatment of schizophrenia patients, but the role of psychosocial variables in determining QoL is still ambiguous. Therefore, in the present research, demographic, clinical and psychosocial variables were examined for their influence on QoL. **Methods:** We conducted a prospective and cross-sectional study on a sample of 139 patients with schizophrenia (72.7% females, age 48.17 ± 10.22) attending an outpatient service. QoL was measured using Schizophrenia Quality of Life Revision-4 (SQLR4) and internalised stigma, self-esteem, perceived social support, resilience and coping mechanisms were assessed using a battery of standardized self-report scales. **Results:** Female patients and those less adherent to treatment had reduced cognition and vitality QoL. The worst QoL in all domains was observed in patients with a younger age at illness onset and with six or more hospitalizations. Regression analysis indicated that reduced self-esteem, perceived social support, a larger number of hospitalization and increased internalised stigma predicted poorer overall QoL and accounted for 44.9% in the variance in SQLSR4 global score (adjusted R^2^ = 0.449, *p* = 0.046). **Conclusions:** Routine assessment of internalised stigma, self-esteem and perceived social support, in addition to demographic and clinical variables and addressing possible deficits in these areas through personalized intervention, could improve QoL in schizophrenia patients.

## 1. Introduction

Schizophrenia is a severe and complex mental illness characterized by distortions in thinking, perception, affect and behaviour, as well as cognitive impairments [1]. It affects approximately 1 in 300 persons worldwide and is ranked among top 10 causes of disability [2,3]. Despite its low lifetime prevalence, schizophrenia imposes a substantial health, social and economic burden on patients, their families, caregivers and society as a whole [4]. Patients encounter varying degrees of disability across multiple functional areas [5] and have a poorer quality of life (QoL) compared to the general population, as shown by meta-analytic evidence [6].

QoL is a broad concept that can be defined from a two-faceted perspective. From a subjective point of view, the World Health Organization defines QoL as “an individual’s perception of his or her position in life in the context of the culture and value systems in which he or she lives, and in relation to his or her goals, expectations, standards, and concerns” [7]. Objectively, QoL is measured through various living, financial and social indicators [8]. Ritsner M. (2007) argues that impairments in health-related QoL in schizophrenia form a distinct syndrome that is influenced by multiple factors. These factors may have a protective or distressing effect [9]. A wealth of research on factors affecting QoL has been focused on the role of symptomatology. In this context, although a meta-analysis revealed a small negative association between QoL and psychiatric symptoms [10], a systematic review found that positive or depressive symptoms may not influence QoL [11]. Also, some studies have reported different demographic and clinical factors (i.e., marital status, monthly household income, employment, living arrangement, level of education, number of hospitalizations, age, illness duration, adherence to medication, body mass index—BMI, etc.) as predictors of QoL [12,13,14,15,16,17,18,19,20,21,22]. Furthermore, while these studies show inconsistent results, other research has failed to identify any demographic or clinical characteristics as potential predictors of QoL in schizophrenia [23]. Therefore, clinical and demographic variables seem to have a small influence on QoL among schizophrenia patients. For this reason, researchers recommend exploring factors beyond the medical model to gain a better understanding of health-related QoL [24,25]. In addition, these inconsistent results regarding clinical and demographic factors call for future research to deepen our understanding of their actual contribution in determining QoL.

Psychosocial factors associated with emotional regulation, such as social support, feelings of self-esteem, resilience, internalised stigma and coping mechanisms to stressful situations, are presumed to be involved in predicting QoL, as they may serve as protective factors, according to the Ritsner’s distress/protection vulnerability model of QoL outcome [9]. Moreover, The Substance and Mental Health Service Administration National Consensus Statement on Mental Health Recovery proposed 10 recovery components that include the above-mentioned self-factors. Numerous researchers have recognised that the term recovery is similar to the concept of QoL [26]. Broadly speaking, stigma is regarded as one of the most significant challenges encountered by people with psychosis. A recent meta-analysis revealed that high levels of internalised stigma have been linked to lower subjective QoL [27]. Similar results were reported concerning self-esteem [28]. Also, studies showed that self-esteem mediates the relationship between different clinical and psychological outcomes (i.e., depression, stigma, and medication compliance) and QoL [29,30,31], further emphasizing the important role of self-esteem in determining QoL in schizophrenia. Social support is thought to be a crucial factor in the course of many mental health illnesses by attenuating stress or increasing self-esteem [32]. In schizophrenia, it was revealed that social support is associated not only with symptom improvement or even remission, as well as fewer hospitalizations, but also with a better overall QoL [16,33,34,35,36,37,38,39,40]. Unfortunately, there are limited data on this topic.

Schizophrenia patients encounter considerable challenges and stress due to positive symptoms, cognitive deficits and decreased functionality [41]. Resilience is the ability to adapt in the face of adversity, and it is considered that it may have an important role in achieving and sustaining full recovery in schizophrenia [42]. Current evidence from a systematic review shows that higher resilience in patients with schizophrenia was associated with better quality of life [41]. Another important aspect related to an individual’s stress response is the coping mechanisms they use. “Coping” refers to the conscious and unconscious psychic mechanisms that people employ to adaptively manage stress. Individuals use a variety of coping mechanisms to manage or overcome stressful situations, including dealing with the wider impact of mental illness [43]. As noted by Kao et al. (2017) and Montemagni et al. (2014), people with schizophrenia have difficulties in coping with life stresses and generally use less effective coping strategies than healthy individuals [44,45]. Regarding QoL, Yanos and Moos (2007) proposed coping as a major determinant [46].

However, as previously noted, limited research has explored all these factors simultaneously to assess their impact on QoL. Therefore, it is imperative to deepen our comprehension of their intricate relationships and contributions to defining QoL. Thus, this study aimed to investigate the influence of various demographic and clinical factors as well as internalised stigma, social support, self-esteem, coping strategies and resilience on predicting QoL in patients with schizophrenia. Understanding the factors that affect QoL in patients with schizophrenia could aid in creating targeted interventions to improve their QoL.

## 2. Materials and Methods

### 2.1. Study Design, Procedures and Population

We conducted a prospective, cross-sectional and observational research study from 1 July 2022 to 31 December 2023, on a sample of 139 patients with schizophrenia. Patients were recruited from the outpatient units and the Mental Health Centre of the “Prof. Dr. Alexandru Obregia” Clinical Hospital of Psychiatry in Bucharest, Romania. The study was conducted in accordance with the ethical principles of the Declaration of Helsinki for Medical Research involving human subjects and was approved by the Institutional Ethics Committee (approval no. 90/07.06.2022). All patients provided written informed consent before entering the study.

Participants were recruited during regular appointments to their treating psychiatrist. Patients were included in this study if they met the following criteria: (1) age between 18 and 65 years old; (2) have a diagnosis of schizophrenia according to the 10th revision of the International Classification of Diseases criteria confirmed by their treating psychiatrist based on a semi-structured interview and the patient’s history; (3) clinically stable and with adequate symptom control; the patient is not in an acute episode and has more than 3 months after the last hospitalization or major change in treatment regimen; (4) the patient agreed to participate in the study and signed the informed consent. We applied the following exclusion criteria: (1) presence of a serious medical illness; (2) patients with first-episode schizophrenia; (3) presence of a neurological or organic cerebral pathology; (4) mental retardation; (5) psychiatric comorbidities (including addition to alcohol or other psychoactive substances, except nicotine); (6) uncorrected visual or hearing impairments that may influence understanding of the information presented or performance in completing a self-administered scale; (7) pregnancy or lactation; (8) inability to follow the instructions; (9) the patient did not give his consent to participate in the study and/or did not sign the informed consent form.

Each patient was asked to provide socio-demographic and clinical data and to complete self-administered scales assessing quality of life, internalised stigma, perceived social support, coping mechanisms, resilience, self-esteem and adherence to treatment.

### 2.2. Measures

#### 2.2.1. Socio-Demographic and Clinical Variables

We recorded the following socio-demographic and clinical data for each patient using a semi-structured questionnaire especially designed for this research: age (years), gender (male or female), residence (urban or rural), marital status (with partner or single), years of formal education (≤8, 9–12, >12 years), residential status (lives alone or lives with other—family, friends, partner), professional status (unemployed, disability pension, retirement pension, employed), income source (personal income, family support, personal income and family support), duration since last hospitalization (months), age at illness onset (years), duration of illness (years), number of hospital admissions (none, 1, 2–5, ≥6), height (cm), weight (kg), type of antipsychotic administration (oral, long-acting injectable—LAI, oral and LAI), type of antipsychotic (typical, atypical, combination of typical and atypical), presence of somatic comorbidities.

#### 2.2.2. Instruments

After collecting socio-demographic and clinical data, each participant was given a set of self-administered psychometric scales that contained the following: Schizophrenia Quality of Life Revision 4 (SQLR4), Internalised Stigma of Mental Illness (ISMI), Multidimensional Scale of Perceived Social Support (MSPSS), Coping Orientation to Problems Experience (COPE) Questionnaire, Brief Resilience Scale (BRS), Rosenberg Self-Esteem Scale and Medication Adherence Rating Scale (MARS).

The SQLSR4 was used to evaluate patient’s quality of life. This disease-specific self-administered instrument includes 33 items and was specifically designed to measure quality of life from the patient’s perspective. All items, except for four, are scored on a 5-point Likert scale from 0 to 4. The scale comprises 2 domains, specifically “psychosocial feelings” (20 items) and “cognition and vitality” (13 items). The global score and the two subscale scores are transformed to have a range from 0 to 100, and a lower score represents a better quality of life [47]. The SQLR4 has been extensively used in studies assessing QoL of schizophrenia patients [48,49,50] and has proven, good psychometric proprieties [51,52,53,54]. In our study, the Romanian version of the SQLSR4 demonstrated good internal consistency (Cronbach’s alpha = 0.93).

The ISMI is one of the most-used measures of subjective experience of stigma and assesses five thematic areas (i.e., alienation, stereotype endorsement, perceived discrimination, social withdrawal and stigma resistance). It comprises 29 items rated on a 4-point Likert scale (from “strongly disagree” to “strongly agree”). A higher score is indicative of a more severe self-stigma [55]. Although originally designed for a broad spectrum of mental illnesses, the scale was applied to samples of patients with schizophrenia, including those from Romania [56]. The Romanian version is available and was produced within the GAMIAN-Europe study [57,58]. In this research, the Romanian version of ISMI had good internal consistency, with a reliability coefficient alpha of 0.87.

The COPE Questionnaire was developed by Carver et al. (1989) to evaluate how people respond to challenging or stressful situations in their lives [59]. The original instrument comprises 53 items that encompass 14 strategies [59], which can be further categorised as either adaptive or maladaptive [44]. The following domains are considered adaptive: positive reinterpretation, venting of emotions, use of instrumental social support, active coping, religious coping, restraint, use of emotional social support, acceptance, suppression of competing activities and planning. Mental disengagement, denial, behavioural disengagement and alcohol–drug use are viewed as maladaptive coping mechanisms [44]. The scale was translated into Romanian and underwent psychometric validation [60,61]. The COPE has been also used in clinical samples, specifically those with schizophrenia-spectrum disorders [62,63]. Internal consistency in our study was satisfactory for the 14 subscales (Cronbach’s alpha ranging from 0.70 to 0.79), as well as for the two coping domains (Cronbach’s alpha of 0.88 for adaptive coping and Cronbach’s alpha of 0.73 for maladaptive coping).

Developed by Zimet et al. (1988), the MSPSS aims to capture the self-perceptions of the support received from friends, family and significant others. The instrument is short and consists of 12 statements, divided into four for each source of support. Respondents are asked to rate each statement on a scale ranging from 1 (strongly disagree) to 7 (strongly agree) [64,65]. The scale has been previously translated into Romanian and proved to be valid and reliable [66,67]. Although initially developed on undergraduate students [64], the MSPSS has been shown to be suitable for assessing perceived social support among patients with schizophrenia [68]. In the current study, all three subscales and the total scale demonstrated excellent internal reliability (Cronbach’s alpha = 0.874, 0.940, 0.921 and 0.859, respectively).

The Rosenberg Self-Esteem scale is regarded as the benchmark for measuring self-esteem. It is a 10-item questionnaire, and responses are rated on a 4-point Likert scale. Higher scores correspond to increased self-esteem levels [69]. The instrument has been utilized in several studies involving patients with schizophrenia [70,71], and its validity and reliability have been confirmed for the Romanian version [60].

The BRS is a 6-item measure that assesses an individual’s capacity to bounce back or recover from stress. The BRS total score ranges from 6 to 30, where higher scores reflect greater resilience [72]. The BRS was validated to be used in people with serious mental illnesses (including schizophrenia) [73]. The scale was translated and adapted for the Romanian population, and it showed good internal consistency across several studies [74,75,76]. In the current study, the BRS yielded a Cronbach’s alpha of 0.74.

The MARS is intended to measure the level of psychoactive medication compliance and was developed by Thompson et al. (2000) [77]. Respondents are instructed to rate each statement with a score of 0 or 1. The total score is calculated by summing the scores of all items, with a higher total score indicating better compliance [29,77]. In the current study, the internal consistency of the instrument, as measured by Cronbach’s alpha, was 0.77.

### 2.3. Statistical Analysis

The statistical analysis was computed with IBM Statistical Package for Social Sciences (SPSS) version 26.0. software for Windows (IBM, Armonk, NY, USA). Sample size calculation was based on the primary outcome: identification of independent predictors associated with SQLSR4 in patients with schizophrenia. The parameters taken into consideration for sample size calculation were an alpha error of 0.05, a beta error of 0.8 (study power), an effect size (F) of 0.39 (medium effect) and a number of 15 possible predictors identified in the literature. Thus, a number of 137 subjects should be included in the entire regression model in order to have a statistical power of 0.8015. Categorial variables were expressed as absolute (number) and relative (percentage) frequency, and continuous variables were expressed as mean and standard deviation (mean ± standard deviation). Continuous data from two independent groups were compared using the independent t-test, and for three or more groups, ANOVA (analysis of variance) was employed. Bivariate correlations between continuous variables were performed using Pearson’s correlation coefficients. A stepwise forward method was used to build multivariate regression models. Factors were kept in the model if probability of F to enter was ≤0.05 and removed if probability of F to remove was ≥0.1. Separate models were built taking the following as the dependent variable: SQLS-R4 psychosocial score, SQLS-R4 cognition and vitality score, and SQLS-R4 global score. The sociodemographic, clinical and psychosocial variables were tested for independent predictive value. Effect size was expressed as adjusted R square. *p* values less than an alpha level of 0.05 were considered to be statistically significant.

## 3. Results

One hundred and eighty-four patients were invited to participate in the study, and one hundred and fifty agreed to participate and signed the informed consent. A total of 11 patients were excluded based on the inclusion and exclusion criteria (age >65 years old). One hundred and thirty-nine schizophrenia patients were included in the final analysis in this research. The mean (±SD) age of the participants was 48.17 (±10.22), and 72.7% of them were female. Most of the patients lived in urban areas (79.9%) with someone (family, friends or partner) (80.6%). The mean (±SD) duration of illness was 23.95 (±9.06), and more than half of the participants had six or more hospital admissions (53.2%). All sociodemographic and clinical data are presented in Table 1.

Only the SQLS cognition and vitality score was associated with gender (female vs. male: 38.78 ± 10.88 vs. 34.36 ± 13.32, *p* = 0.048). SQLS total, SQLS psychosocial and SQLS cognition and vitality were associated with age at illness onset (R = −0.206, *p* = 0.015; R = −0.177, *p* = 0.037; and R = −0.226, *p* = 0.007, respectively) and number of hospital admissions (2–5 admissions vs. >6 admissions: 32.33 ± 12.07 vs. 39.58 ± 10.73, *p* = 0.000; 31.71 ± 13.47 vs. 38.46 ± 11.98, *p* = 0.000; and 33.28 ± 11.38 vs. 41.32 ± 10.74, *p* = 0.000, respectively). The MARS score was negatively associated with the SQLS cognition and vitality score (R = −0.167, *p* = 0.049). The other socio-demographic and clinical characteristics of the study sample were not associated with any of the SQLS scores (*p* > 0.05) (Table 1).

Patients had a mean (±SD) SQLS total score of 36.19 (±11.90). The means (±SD) for the psychosocial and cognition and vitality subscales were 35.30 (±13.10) and 37.56 (±11.72), respectively. Regarding stigma, the sample had a mean (±SD) for the global ISMI score of 2.41 ± 0.23, which indicates mild internalised stigma. For the MSPSS, the mean (±SD) score was 4.35 ± 0.70. The group had normal resilience levels (3.00 ± 0.20), and the mean (±SD) of the self-esteem score was 27.44 ± 3.16. Table 2 presents all results regarding psychometric scales scores.

Bivariate analyses found that the ISMI showed significant positive correlations with the overall QoL score and both domains (*p* < 0.01). The MSPSS and Rosenberg Self-Esteem negatively correlated with all SQLS scores (*p* < 0.01) (Table 3).

For global QoL and for each QoL domain a distinct predictive model was found using stepwise multiple regression analysis (Table 4). The Rosenberg Self-Esteem score, internalised stigma, social support and number of hospitalizations explained 44.9% of the variance (adjusted R^2^) in the SQLS global score. More precisely, higher self-esteem, fewer hospital admissions, lower internalised stigma and greater perceived social support predicted a better overall QoL. The psychosocial domain score was predicted by the Rosenberg Self-Esteem score, MSPSS score and number of hospitalizations. This model explained 38.9% of the variance. Also, the model including the Rosenberg Self-Esteem score, ISMI score and the number of hospitalizations explained 42.5% of the variance in the SQLS cognition and vitality score (Table 4).

## 4. Discussion

The current study sought to identify demographic, clinical, and psychosocial determinants of QoL in a sample of outpatients with schizophrenia. Therefore, it was demonstrated that increased self-esteem and perceived social support, lower internalised stigma and fewer hospital admissions independently predicted better global QoL. These factors explain up to 44.9% of the variance in overall QoL. Higher self-esteem and fewer hospitalizations continued to be significant predictors for both QoL domains. Greater perceived social support and reduced self-stigma, specifically, predicted the psychosocial and cognition and vitality domains of QoL, respectively.

SQLS scores in our sample are comparable to those found in other studies involving schizophrenia outpatients, inpatients, or both [47,49,51,52,54,78,79], even though the mean SQLS global score in our study is lower than it was estimated by a recent meta-analysis (40.66, 95% CI: 37.66–43.66) [80]. Moreover, based on experts’ opinions on the range of SQLS-R4 scores (very good: 0–20, good: 21–40, moderate: 41–60, poor: 61–80, very poor: 81–100), the global QoL in our sample (36.19 ± 11.90) can be categorised as good to moderate [80].

### 4.1. Demographic and Clinical Variables and Quality of Life

Findings on the relationship between socio-demographic variables and QoL measured using SQLS-R4 have been inconsistent and conflicting. In the present study, among all demographic variables, only gender was found to have a relationship with QoL; specifically, women had poorer QoL in the cognition and vitality domain compared to men. Similar results were reported by Domenech et al. (2019) for the global score of SQLS—original version [81] and by other researchers using a generic QoL scale [82], but results at difference with these are reported in the current literature [83,84]. The large female representation in our sample (72.7%) may be a contributing reason for this association. Moreover, individuals with an older age at schizophrenia onset and those with fewer hospitalizations had a better QoL than the other patients in our study, regarding all components of SQLS-R4. In addition, a lower number of hospital admissions predicted a better QoL. Zhou et al. (2024) reported findings consistent with ours concerning age at illness onset but not number of hospitalizations [85]. Age at illness onset is of outmost importance in understanding the course of schizophrenia. It significantly influences the course of the illness, with early onset being associated with a more severe prognosis, including more negative symptoms, lower educational attainment, reduced employability, impaired social functioning and a poorer global outcome [86]. These observations might explain our results where a younger age at onset was related to worse QoL even though other studies provide different results [87]. The number of hospitalizations is viewed as a proxy of clinical and functional outcomes, which intersects with concepts such as quality of life and recovery [88]. Using SQLS-R4, Taha et al. (2024) and Kuo et al. (2009) observed that patients with a higher level of education had a poorer global QoL [89,90], and Arraras et al. (2018) found that unemployment was associated with lower QoL in the vitality and cognition domain [49]. We might have not obtained similar results due to the high proportion of patients in our research receiving a disability pension (80.6%) and having 9–12 years of education (81.3%). Other studies failed to show an association between socio-demographic or clinical variables and SQLS-R4 global and/or sub-domain scores [48,50].

### 4.2. Psychosocial Variables and Quality of Life

In the present research, internalised stigma was observed to be correlated with QoL in all domains and, furthermore, to be a predictor of QoL. More precisely, high levels of internalised stigma predicted a poorer global and cognition and vitality QoL. This association aligns with a prior, very limited, body of research utilizing SQLS-R4 to assess QoL, which documented an inverse relationship between QoL and internalised stigma as well as internalised stigma as a predictor of QoL [29,31,50]. These findings were confirmed by a meta-analysis which included studies carried out using SQLS-R4 or SQLS [27]. The explanation for our results derives from data showing that self-stigma affects one’s sense of self (self-esteem and self-efficacy) [91]. Morgades-Bamba et al. (2019) and Kim et al. (2019) showed that internalised stigma decreases QoL through lowering the positive self-concept (i.e., self-esteem) [31,92]. Moreover, self-prejudice and self-discrimination lead not only to a negative emotional reaction (i.e., lower self-esteem) but also to a negative behavioural response (e.g., neglecting to seek employment or housing opportunities and becoming socially isolated) that interferes with QoL [93]. These observations also align with our findings on the influence of self-esteem on quality of life. Specifically, our study demonstrated that lower self-esteem is associated with poorer quality of life in all aspects and identified self-esteem as an independent predictor of life satisfaction. Studies by Morgades-Bamba et al. (2019), Kim et al. (2019) and Lien et al. (2018) using SQLS-R4 or SQLS have demonstrated similar results [31,92,94]. In addition, a recent meta-analysis showed that there is a strong negative correlation between self-esteem and QoL in individuals with schizophrenia spectrum disorders [28]. However, one possible explanation for our results could be the overlap between the construct of the SQLSR4 psychosocial domain and Rosenberg Self-Esteem scale [79].

Current theories suggest that coping with life stress strategies employed by persons with schizophrenia influence their QoL [46,95]. Patients with schizophrenia have been observed to be more likely to adopt maladaptive or negative coping strategies, exhibit a lower use of adaptive coping, or perceive their coping strategies as less effective than healthy controls [43,96,97,98,99]. Holubova et al. (2015) identified a correlation between negative coping strategies and poorer overall quality of life in patients with schizophrenia, as well as specific quality of life subdomains, including physical health, emotional well-being, work and social activities [43]. Ritsner et al. (2003b) and Montemagni et al. (2014) showed that a social diversion coping strategy (i.e., strategy based on seeking social support) positively influenced QoL [45,100], with coping accounting for up to 25% in the variance of QoL [100]. Navarro et al. (2018) revealed that problem-focused coping (i.e., planning, active coping and suppression of competing activities, which are considered adaptive), but not avoidance coping (i.e., behavioural disengagement, denial, substance use and mental disengagement, which are considered maladaptive), predicted QoL (i.e., physical, psychological and environmental QoL domains) [101]. In contrast, Bechdolf et al. (2003) showed that negative coping (i.e., social withdrawal, resignation and self-pity) was a predictor of poorer QoL [40]. Our results are in agreement with previous data and show that use of maladaptive coping strategies (i.e., mental disengagement, denial, behavioural disengagement and alcohol–drug use) was associated with poorer global and psychosocial QoL. Future studies assessing coping strategies in persons with schizophrenia could shed more light on the relationship between coping and QoL.

The social support of schizophrenia patients became of great interest after the deinstitutionalization process, being instrumental to the successful adaptation of patients in the community and rehabilitation [102]. Additionally, social support is believed to act as a buffer between the patient and various stressors, playing a significant role in the vulnerability–stress model of disease [37]. Unfortunately, perceived social support is not well studied in schizophrenia patients. Munikanan et al. (2017) researched the impact of perceived social support measured using the MSPSS on QoL. It was reported that perceived social support predicted better QoL in all domains (i.e., physical, psychological, social and environmental) [38]. Similar results were observed by other researchers [16,39,40,103]. In addition, Ritsner et al. (2012) showed that an increase in the perceived support from a significant other or friends predicted better QoL over a 10-year follow-up period [34]. Our findings provide evidence in line with previous research and show that perceived social support was associated with all domains of quality of life and demonstrated predictive ability for overall QoL and, of course, the psychosocial domain. Specifically, higher levels of perceived social support were linked to improved quality of life.

Adherence to antipsychotic medication is a challenging issue in schizophrenia and has an important impact on the course of illness. Non-adherence leads to relapse and frequent hospitalizations, which corresponds to worse functional outcomes and delayed recovery [104,105,106,107]. Yu et al. (2021) found that both the psychosocial and cognition and vitality domains were influenced by treatment adherence, measured using MARS [108]. Other researchers found that adherence to medication was a predictor of better QoL [13,106], but results at difference with this are reported in the current literature [109]. Moreover, improvement in QoL over the course of one year was predicted by improvement in treatment adherence [14]. In our study, the MARS score was not a predictor of QoL, but we found a significant negative relationship between the SQLS vitality and cognition score and MARS score, meaning that better adherence to treatment is associated with better QoL. An explanation for our results comes from the inclusion of chronic community dwelling schizophrenia patients, who demonstrated strong adherence to the prescribed treatment.

### 4.3. Implications for Future Practice and Study Limitations

Our findings have important implications for outpatient care in schizophrenia. Our results support the need for tailored psychosocial interventions alongside antipsychotic therapy in order to improve QoL, which is increasingly recognized as a key treatment outcome in outpatients with schizophrenia. The majority of psychosocial interventions have been primarily focused on symptom reduction or functioning and overshadowed the importance of QoL and well-being [110,111]. Improving QoL in people with schizophrenia by incorporating psychological interventions successfully proven to reduce self-stigma and improve treatment adherence is recommended [112]. In this regard, Sibitz et al. (2013) showed that a recovery-oriented programme that covered self-stigma and compliance to treatment aspects had a positive impact on QoL as well [113].

Even though our study contributes to the current understanding of predictors of QoL in patients with schizophrenia, it has several limitations that should be acknowledged. Firstly, the number of patients included is relatively low, and studies with larger samples are warranted in order to confirm our results. Also, our study included stable outpatients, thus the results lack generalizability to patients treated in other settings. Another limitation is that symptom severity, which may affect QoL, was not evaluated; however, our study focused on exploring demographic and psychosocial aspects involved in shaping QoL. Fourthly, this researched was carried out using a cross-sectional design, thus it prevented us from establishing causal relationships between variables. Also, SQLSR4 had moderate to good correlations with the Rosenberg Self-Esteem scale, which indicates several construct similarities despite their distinctive purposes; therefore, this might be another limitation of this study. Finally, we used a subjective assessment of QoL, thus objective measures could lead to different results.

## 5. Conclusions

Our study acknowledges self-esteem, perceived social support and internalised stigma as the most important psychosocial variables that affect subjective QoL in patients with schizophrenia. More precisely, 44.9% in the variance in QoL was predicted by these factors, alongside the number of previous hospitalizations. Since psychosocial interventions have been found to be superior in improving QoL in comparison to antipsychotic treatment alone [114], comprehensive assessment of patients suffering from schizophrenia and then addressing these aspects through integrated and tailored psychosocial therapies seems the most promising measure to improve patients’ QoL.

## Figures and Tables

**Table 1:** Socio-demographic and clinical characteristics of the analysed sample and their correlations with SQLS-R4 scores.

Patient Characteristics	Mean ± SD or N (%)	SQLS-R4 Global		SQLS-R4 Psychosocial		SQLS-R4 Cognition and Vitality	
		Mean ± SD	*p* (R)	Mean ± SD	*p* (R)	Mean ± SD	*p* (R)
**Age**	48.17 ± 10.22		0.12 (−0.132)		0.09 (−0.145)		0.28 (−0.091)
**Gender**							
Female	101 (72.7%)	37.39 ± 11.64	0.05	36.50 ± 13.10	0.07	38.76 ± 10.88	0.048
Male	38 (27.3%)	32.99 ± 12.13		32.10 ± 12.69		34.36 ± 13.32	
**Residence**							
Urban	111 (79.9%)	36.84 ± 12.15	0.19	35.81 ± 13.56	0.36	38.44 ± 11.48	0.07
Rural	28	33.60 ± 10.68		33.30 ± 11.04		34.06 ± 12.19	
**Marital status**							
Single	66 (47.5%)	35.81 ± 11.85	0.72	35.49 ± 13.49	0.85	36.91 ± 12.36	0.53
With partner	73 (52.5%)	36.54 ±12.01		35.09 ±12.74		38.14 ± 11.15	
**Level of education**							
≤8 years	20 (14.4%)	35.90 ± 10.14	0.87	35.43 ± 11.90	0.80	36.63 ± 8.38	0.91
9–12 years	113 (81.3%)	36.11 ± 12.32		35.09 ± 13.46		37.67 ± 12.30	
>12 years	6 (4.3%)	38.63 ± 10.56		38.75 ± 11.06		38.46 ± 11.40	
**Living situation**							
Alone	27 (19.4%)	35.32 ± 11.39	0.67	35.23 ± 12.67	0.97	35.47 ± 11.16	0.30
With others (family, friends, partner)	112 (80.6%)	36.40 ± 12.06		35.32 ±13.25		38.06 ± 11.84	
**Professional status**							
Unemployed	10 (7.2%)	40.22 ± 6.80	0.41	40.50 ± 7.97	0.42	39.80 ± 7.90	0.34
Disability pension	112 (80.6%)	35.36 ± 12.45		34.44 ± 13.69		36.77 ± 12.05	
Retirement pension	6 (4.3%)	39.89 ± 11.81		36.87 ± 11.08		44.55 ± 14.25	
Employed	11 (7.9%)	38.98 ± 9.04		38.52 ± 10.95		39.68 ± 8.90	
**Source of income**							
Personal income	77 (55.4%)	36.65 ± 10.96	0.50	35.61 ± 12.61	0.69	38.26 ± 10.08	0.29
Family support	15 (10.8)	38.43 ± 8.91		37.25 ± 10.66		40.25 ± 8.94	
Family support and personal income	47 (33.8%)	34.71 ± 14.07		37.17 ± 14.64		35.55 ± 14.57	
**Age at illness onset (years)**	24.22 ± 5.15		0.01 (−0.206)		0.03 (−0.177)		0.007 (−0.226)
**Illness duration (years)**	23.95 ± 9.06		0.71 (−0.031)		0.46 (−0.062)		0.76 (0.026)
**Time since last hospital admission (months)**	15.34 ± 27.12		0.778 (0.24)		0.827 (0.019)		0.727 (0.030)
**Number of hospital admissions**							
2–5	65 (46.8%)	32.33 ± 12.07	0.000	31.71 ± 13.47	0.000	33.28 ± 11.38	0.000
≥6	74 (53.2%)	39.58 ± 10.73		38.46 ± 11.98		41.32 ± 10.74	
**BMI**	26.89 ± 5.95		0.70 (0.032)		0.70 (0.032)		0.75 (0.026)
**Type of antipsychotic administration**							
Oral	82 (59%)	36.98 ± 11.93	0.63	35.77 ± 13.04	0.87	38.83 ± 11.67	0.24
LAI	35 (25.2%)	35.34 ± 10.89		34.53 ± 11.95		36.59 ± 10.74	
Oral and LAI	22 (15.8%)	34.60 ± 13.54		34.77 ± 15.41		34.35 ± 13.08	
**Type of antipsychotic**							
Typical	12 (8.6%)	36.61 ± 10.53	0.97	35.20 ± 12.24	0.99	38.78 ± 8.78	0.78
Atypical	118 (84.9%)	36.20 ± 11.53		35.28 ± 13.02		37.61 ± 11.85	
Typical and atypical	9 (6.5%)	35.53 ± 14.13		35.69 ± 16.50		35.25 ± 14.16	
**Adherence to treatment (MARS score)**	9.01 ± 1.13		0.18 (−0.112)		0.40 (−0.071)		0.049 (−0.167)
**Presence of somatic comorbidities (no)**							
No	135 (97.1%)	36.18 ± 11.97	0.95	35.33 ± 13.13	0.88	37.49 ± 11.83	0.68
Yes	4 (2.9%)	36.55 ± 10.57		34.37 ± 13.71		39.90 ± 7.75	

N, number of patients; SD, standard deviation; LAI, long-acting antipsychotic; BMI, body mass index; SQLS-R4, Schizophrenia Quality of Life Scale—Revision 4; MARS, Medication Adherence Rating Scale.

**Table 2:** Psychometric scales scores.

Measure	Score (Mean ± SD)
SQLS global	36.19 ± 11.90
SQLS Psychosocial	35.30 ± 13.10
SQLS Cognition and Vitality	37.56 ± 11.72
ISMI total	2.41 ± 0.23
MSPSS total	4.35 ± 0.70
COPE Adaptative coping	2.47 ± 0.30
COPE Maladaptive coping	2.07 ± 0.38
Brief Resilience Scale	3.00 ± 0.20
Rosenberg Self-Esteem	27.44 ± 3.16

SD, standard deviation; SQLS, Schizophrenia Quality of Life; ISMI, Internalised Stigma of Mental Illness; MSPSS, Multidimensional Scale of Perceived Social Support; COPE, Coping Orientation to Problems Experience.

**Table 3:** Correlations coefficients between SQLS scores and internalised stigma, social support, coping strategies, resilience and self-esteem scores.

	SQLS Total	SQLS Psychosocial	SQLS Cognition and Vitality
ISMI	0.331 **	0.285 **	0.362 **
MSPSS	−0.403 **	−0.401 **	−0.348 **
COPE Adaptative coping	0.104	0.173 *	−0.028
COPE Maladaptive coping	0.226 **	0.276 **	0.108
Brief Resilience Scale	0.030	0.068	−0.040
Rosenberg Self-Esteem	−0.615 **	−0.587 **	−0.575 **

SQLS, Schizophrenia Quality of Life; ISMI, Internalised Stigma of Mental Illness; MSPSS, Multidimensional Scale of Perceived Social Support; COPE, Coping Orientation to Problems Experience; *, *p* < 0.05; **, *p* < 0.01.

**Table 4:** Summary of models predicting quality of life: findings from the stepwise multiple linear regressions.

Independent Variable	B	Standard Error	β	t	*p*-Value	Model’s Proprieties
**SQLS global score** (dependent variable)						
Rosenberg Self-Esteem	−1828	0.265	−0.486	−6.891	0.000	adj. R^2^ = 0.449 *p* = 0.046
Number of hospital admissions	4.796	1.525	0.202	3.146	0.002	
ISMI	6.986	3.356	0.140	2.081	0.039	
MSPSS	−2.427	1.205	−0.143	−2.013	0.046	
**SQLS psychosocial score** (dependent variable)						
Rosenberg Self-Esteem	−2.007	0.303	−0.485	−6.624	0.000	adj. R^2^ = 0.389 *p* = 0.02
MSPSS	−3.554	1.360	−0.190	−2.613	0.010	
Number of hospital admissions	4.164	1.766	0.159	2.358	0.020	
**SQLS cognition and vitality score** (dependent variable)						
Rosenberg Self-Esteem	−1.759	0.251	−0.047	−7.007	0.000	adj. R^2^ = 0.425 *p* = 0.002
Number of hospital admissions	5.892	1.531	0.252	3.848	0.000	
ISMI	10.563	3.286	0.216	3.214	0.002	

SQLS, Schizophrenia Quality of Life Scale; ISMI, Internalised Stigma of Mental Illness; MSPSS, Multidimensional Scale of Perceived Social Support; adj., adjusted.

## Data Availability

The data presented in this study are available on reasonable request from the corresponding author. The data are not publicly available due to ethical and institutional reasons.
